# Supplementary material for: A Cross-Sectional Evaluation of Caregiver Burden in Schizophrenia Care: Findings from Western Saudi Arabia with Policy Implications for Preventive Mental Healthcare
Source: Healthcare (Basel). 2025 Dec 25;14(1):55. doi: 10.3390/healthcare14010055 (PMC12785843; doi:10.3390/healthcare14010055)
Supplement: Supplementary file 1 [file healthcare-14-00055-s001.zip › Supplementary Table S1.pdf]

Supplementary Table S1. Participants' responses in each item of the caregiver burden scale (n = 330)

Variable	Never	Rarely	Sometimes	Frequently	Nearly always
Do you frequently feel emotionally worn out from managing caregiving tasks?	37 (11.2)	62 (18.8)	69 (20.9)	76 (23.0)	86 (26.1)
Are there moments when you feel frustrated or upset while caring for your loved one?	33 (10.0)	68 (20.6)	98 (29.7)	70 (21.2)	61 (18.5)
Do you find yourself feeling unsure or uneasy about decisions related to caregiving?	45 (13.6)	51 (15.5)	97 (29.4)	66 (20.0)	71 (21.5)
How often do you feel emotionally strained while spending time related to caregiving?	32 (9.7)	54 (16.4)	65 (19.7)	87 (26.4)	92 (27.9)
Do you feel concerned about your ability to meet your caregiving responsibilities?	30 (9.1)	60 (18.2)	85 (25.8)	80 (24.2)	75 (22.7)
Has your personal time been significantly affected by caregiving duties?	36 (10.9)	65 (19.7)	72 (21.8)	86 (26.1)	71 (21.5)
Do you find it difficult to make time for activities or hobbies you once enjoyed?	42 (12.7)	49 (14.8)	80 (24.2)	77 (23.3)	82 (24.8)
Have caregiving responsibilities limited your ability to connect with friends or family?	43 (13.0)	59 (17.9)	82 (24.8)	64 (19.4)	82 (24.8)
Do you avoid inviting people over because of your caregiving situation?	48 (14.5)	47 (14.2)	83 (25.2)	87 (26.7)	65 (19.7)
Are you struggling to balance caregiving with other roles, such as work or family?	44 (13.3)	58 (17.6)	76 (23.0)	88 (26.7)	64 (19.4)
Has caregiving caused tension in your relationships with family or close friends?	38 (11.5)	70 (21.2)	92 (27.9)	72 (21.8)	58 (17.6)
Do you feel uneasy about how your loved one’s behavior is perceived by others?	52 (15.8)	76 (23.0)	98 (29.7)	42 (12.7)	62 (18.8)

Have you noticed that your relative depends on you more than is necessary?	50 (15.2)	68 (20.6)	69 (20.9)	86 (26.1)	57 (17.3)
Do you feel like you're the only person your relative relies on for help?	49 (14.8)	73 (22.1)	80 (24.2)	63 (19.1)	65 (19.7)
Are you worried that your caregiving role has negatively impacted your relationships?	45 (13.6)	57 (17.3)	74 (22.4)	75 (22.7)	79 (23.9)
Has your physical health declined due to the demands of caregiving?	49 (14.8)	59 (17.9)	70 (21.2)	96 (29.1)	56 (17.0)
Do you feel tired or physically exhausted because of caregiving responsibilities?	45 (13.6)	51 (15.5)	76 (23.0)	94 (28.5)	64 (19.4)
Are financial pressures affecting your ability to provide care for your relative?	39 (11.8)	49 (14.8)	86 (26.7)	88 (26.7)	68 (20.6)
Have caregiving duties left you with little time to focus on your own health?	48 (14.5)	63 (19.1)	84 (25.5)	77 (23.3)	58 (17.6)
Do you feel uncertain about how to manage the financial aspects of caregiving?	47 (14.2)	50 (15.2)	74 (22.4)	81 (24.5)	78 (23.6)
Do you feel pressured to meet all your caregiving needs, even if they exceed what's reasonable?	37 (11.2)	60 (18.2)	73 (22.1)	74 (22.4)	86 (26.1)
Are you afraid that you won't be able to continue providing care in the future?	31 (9.4)	54 (16.4)	67 (20.3)	91 (27.6)	87 (26.4)
