# Supplementary material for: A Cross-Sectional Evaluation of Caregiver Burden in Schizophrenia Care: Findings from Western Saudi Arabia with Policy Implications for Preventive Mental Healthcare
Source: Healthcare (Basel). 2025 Dec 25;14(1):55. doi: 10.3390/healthcare14010055 (PMC12785843; doi:10.3390/healthcare14010055)
Supplement: Supplementary file 1 [file healthcare-14-00055-s001.zip › Supplementary Table S2 EFA.pdf]

Supplementary Table S2: Exploratory Factor Analysis

Variable	Emotional Strain	Time and Social Limitations	Interpersonal Relationships and Dependency	Health and Financial Impacts	Caregiving Expectations
Item 1	0.789	-	-	-	-
Item 2	0.672	-	-	-	-
Item 3	0.598	-	-	-	-
Item 4	0.721	-	-	-	-
Item 5	0.838	-	-	-	-
Item 6	-	0.603	-	-	-
Item 7	-	0.744	-	-	-
Item 8	-	0.573	-	-	-
Item 9	-	0.651	-	-	-
Item 10	-	0.709	-	-	-
Item 11	-	-	0.597	-	-
Item 12	-	-	0.562	-	-
Item 13	-	-	0.713	-	-
Item 14	-	-	0.820	-	-
Item 15	-	-	0.647	-	-

Item 16	-	-	-	0.711	-
Item 17	-	-	-	0.774	-
Item 18	-	-	-	0.606	-
Item 19	-	-	-	0.817	-
Item 20	-	-	-	0.692	-
Item 21	-	-	-	-	0.639
Item 22	-	-	-	-	0.714
